# A Validation Approach for Determining Fetal Blood Groups Non-Invasively by High-Sensitive Next-Generation Sequencing

**DOI:** 10.3390/jcm14196812

**Published:** 2025-09-26

**Authors:** Sandra Wienzek-Lischka, Marion Soelter, Annika Froelich, Marion Ernst-Schlegel, Stefan Gattenloehner, Andreas Braeuninger, Ulrich J. Sachs

**Affiliations:** 1 Institute for Clinical Immunology, Transfusion Medicine and Haemostaseology, Justus-Liebig-University Giessen, 35392 Giessen, Germany; marion.soelter@immunologie.med.uni-giessen.de (M.S.); annikafroelich@web.de (A.F.); marion.ernst-schlegel@immunologie.med.uni-giessen.de (M.E.-S.); ulrich.sachs@immunologie.med.uni-giessen.de (U.J.S.); 2 German Center for Feto-Maternal Incompatibility, 35392 Giessen, Germany; 3 Institute for Pathology, Justus-Liebig-University Giessen, 35392 Giessen, Germany; stefan.gattenloehner@patho.med.uni-giessen.de (S.G.); andreas.braeuninger@patho.med.uni-giessen.de (A.B.)

**Keywords:** non-invasive fetal blood group genotyping, next-generation sequencing (NGS), cell-free DNA (cfDNA), fetal and neonatal alloimmune thrombocytopenia (FNAIT), hemolytic disease of the fetus and newborn (HDFN), Integrin beta-3 (*ITGB3*), HPA-1a, anti-HPA-1a, Kell (*KEL*), anti-K

## Abstract

**Introduction:** For pregnant women with a history of fetal and neonatal alloimmune thrombocytopenia (FNAIT) or hemolytic disease of the fetus and newborn (HDFN), prenatal intervention in subsequent pregnancies may be necessary to prevent complications for the fetus. A non-invasive prenatal diagnostic procedure (NIPD) is recommended for fetal blood group genotyping. RT-PCR is used for fetal *RHD* determination as a reliable screening method with high sensitivity and specificity. For other antigens with variants involving single-base substitutions, droplet digital PCR (ddPCR) and next-generation sequencing (NGS) are recommended to reduce the risk of false-negative results. Only NGS offers the possibility of determining the cell-free fetal DNA (cffDNA) fraction in maternal plasma by sequencing additional gene fragments in parallel, but no standard exists for assay validation. **Material and Methods:** A custom-made primer panel was designed to target the common platelet and red cell antigens involved in fetal red cell and platelet incompatibilities, as well as additional anonymous single-nucleotide polymorphism (SNP) targets for use as an internal control. Amplicon-based NGS was carried out using semiconductor sequencing. For HPA-1a (HPA*1A, *ITGB3*) and K (KEL*01.01, *KEL*) assay validation, the limit of detection (LOD) and limit of quantification (LOQ) were estimated, as were false-positive antithetic alleles, linearity, and inter-assay variation, using cell-free DNA (cfDNA) extracted from the blood samples of healthy blood donors. An additional analysis was performed using 23 diagnostic samples from 21 pregnant women. **Results:** Regression analysis of dilution series using HPA-1a- and K-positive cell-free plasma samples in antigen-negative donor plasma showed that recovery is definitely feasible up to an HPA*1A and KEL*01.01 allele frequency of 1%. Base calls of false-positive antithetic alleles were detected with a maximum of 0.25% using 21 healthy blood donors. The LOD was estimated to be 0.2057% (mean + 3 SD) for HPA*1A with a LOQ of 0.6298% (mean + 10 SD). For KEL*01.01, the LOD was 0.1706% (mean + 3 SD) and the LOQ was 0.5314% (mean + 10 SD). The analysis of 15 of 21 cases with diagnostic samples from pregnant women with neonatal blood available for confirmatory testing resulted in 100% concordant results. The fetal fraction of these samples was calculated with a median of 11.03% (95% CI: 8.89, 13.20). **Conclusions:** NGS for non-invasive fetal blood group genotyping is an accurate and reliable method. In-house validation of the used assays can be performed using healthy donors to determine the LOD, LOQ and sensitivity. The threshold for paternally inherited fetal HPA*1A and KEL*01.01 alleles could be set at 1% (i.e., 2% fetal fraction) to obtain reliable test results. Internal controls for assessing the fetal fraction are essential to avoid false-negative test results.

## 1. Introduction

Both fetal and neonatal alloimmune thrombocytopenia (FNAIT) and hemolytic disease of the fetus and newborn (HDFN) are caused by maternal antibodies targeting platelets or red blood cells carrying alloantigens inherited from the father. Invasive fetal diagnostics by chorionic villous sampling or amniocentesis have associated risks and may bolster the maternal alloantibody [1,2]. Fetal genotyping using cell-free fetal DNA in maternal plasma is considered the method of choice [3]. In most Caucasian women, FNAIT is caused by antibodies against the HPA-1a antigen [4]. In HDFN, maternal anti-D antibodies are associated with the highest risk of fetal morbidity and mortality [5]. RT-PCR and digital droplet PCR are the methods of choice for the diagnosis of fetal *RHD* in large cohorts and for screening purposes. These methods are validated and cost-effective for high-throughput testing of different exons of the *RHD* gene and recommendations for validation and quality assurance are published [6]. Diagnostic tests for other less frequent fetal-maternal incompatibilities involved in HDFN and FNAIT, such as Kell or HPA-1a, are established only in a few laboratories. False-negative results cannot be excluded when using RT-PCR for non-invasive genotyping of fetal *HPA* or *KEL* alleles based on single nucleotide exchanges, without internal controls or markers for fetal fraction. For prenatal diagnostics, the targeted amplicon-based NGS approach has the ability to estimate the fetal fraction of cfDNA. Rieneck et al. showed proof of principle using a targeted NGS approach [7]. Our group published an NGS-based fetal HPA-1a typing assay in 2015 [8], followed by NGS-based approaches by Orzińska et al. [9,10,11] and McGowan et al. for a case with HPA-15 incompatibility [12]. Emahazion et al. reported a validation with a prototype kit from a commercial manufacturer [13]. In a more recent publication, droplet digital PCR (ddPCR) and NGS were recommended for NIPD fetal blood group genotyping to reduce false-negative test results. Furthermore, NGS was considered particularly effective in reducing the likelihood of false-negative results [14].

For the validation of *RHD* NIPD assays, a commercially available *RhD/SRY* plasma DNA sensitivity standard (1st WHO Reference Reagent, NISBC) exists, and recommendations for validation have been published [6]. In contrast, no standards are available for other red blood cell or platelet antigens. In cases of fetal–maternal incompatibilities, and in rare cases where the number of samples from pregnant women is insufficient for validation studies of in-house assays, test material is required. In this article, we present a validation approach using spike assays with cfDNA extracted from healthy male blood donors to determine the limit of detection (LOD), limit of quantification (LOQ), and sensitivity.

## 2. Material and Methods

### 2.1. Assay Validation/Subjects

For clinical validation, cell-free DNA from plasma of 21 pregnant women between 13 and 30 weeks of gestation was tested. Fetal genotype was confirmed through postnatal typing of the implicated antigen (sensitivity and specificity). For analytical validation, LOD and LOQ were determined using blood from healthy blood donors. Written informed consent was obtained from all patients and blood donors. The study protocol was approved by the ethics committee of the Medical Faculty, Justus-Liebig-University Giessen, Germany (178/2013 and 05/2000).

### 2.2. Cell-Free (Fetal) DNA Preparation

Blood samples (2 × 10 mL) were collected into blood collection tubes (Cell-Free DNA BCT CE, Streck, La Vista, NE, USA). After centrifugation at 1200× *g* for 10 min at room temperature, the plasma was carefully removed and centrifuged a second time at 2400× *g* for 20 min (validated local protocol according to [8]). The plasma samples were stored at −20 °C until further preparation. Cell-free DNA was extracted from 2 mL of plasma using a magnetic bead binding method (QIAamp MinElute ccfDNA Mini Kit, QIAGEN, Hilden, Germany), lowering the final elution volume to 30 µL. DNA concentration was determined using the Qubit dsDNA HS Assay with the Qubit 2.0 Fluorometer (Life Technologies, Eugene, OR, USA).

### 2.3. Assay Design

An amplicon-based targeted sequencing approach was chosen and the short fragment size of cffDNA was considered in the primer design to reduce the probability of non-call results caused by low fetal fraction [15,16].

The targeted sequences were chosen to flank SNPs/exonic regions encoding common blood group systems of platelets (PLTs) and red blood cells (RBCs) that are frequently involved in FNAIT or hemolytic disease of the newborn (Supplementary Table S1). For estimation of the fetal fraction, several anonymous SNP targets that are unlinked, nonexonic markers that show consistently high minor allele frequency across a diverse group of human populations [17], were included.

### 2.4. Targeted Massively Parallel Sequencing

Semiconductor sequencing with short amplicon-based NGS with UMI was established, performing library preparation according to manufacturer’s instructions (Ion AmpliSeq HD Library Kit, Ion AmpliSeq HD Dual Barcode Kit 1–24, Life Technologies/Thermo Fisher Scientific, Carlsbad, CA, USA) using a custom-made primer panel (Ion AmpliSeq Designer, Life Technologies/Thermo Fisher Scientific, Pleasanton, CA, USA). The Ion AmpliSeq HD Dual Kit contained unique molecular identifiers (UMI). The DNA libraries were quantified by qPCR with the StepOnePlus Real-Time PCR System (Applied Biosystems) using the Ion Library TaqMan Quantitation Kit (Applied Biosystems/Life Technologies, Austin, TX, USA).

The template was prepared with an automated workflow system (Ion ONE Touch system, Life Technologies/Thermo Fisher Scientific). Massively parallel sequencing was performed on semiconductor chips (Ion Torrent 318, Life Technologies/Thermo Fisher Scientific) using a personal genome machine (Ion Torrent PGM platform, Life Technologies/Thermo Fisher Scientific; analysis flow, 500, 125 cycles). A detailed protocol is available upon request from the corresponding author.

### 2.5. Bioinformatics/Statistical Analysis

For bioinformatic analysis, data were processed initially with the Ion Torrent platform-specific software Torrent Suite (Version 5.12.0, Life Technologies/Thermo Fisher Scientific) to generate sequence reads. The Torrent Variant Caller mapping program was used to align the sequence data to the hg19 human reference genome. GraphPadPrism 8.0.2 (263) was used for statistical evaluation.

### 2.6. Definitions and a Priori Defined Cut-Off Values

Total reads for a given locus were defined as the number of all sequence reads that crossed the targeted SNP. Unexpected base calls (UEBCs) were defined as all base calls at a given SNP position that did not belong to the known biallelic system. False-positive antithetic alleles (FPATAs) are signals of a not-estimated base of a biallelic system and known SNP genotype. Sequences were assumed to be of fetal origin if the pregnant woman was homozygous for a given SNP and the antithetical allele at that locus was detected with a frequency of at least 1% of the total reads. The fractional fetal DNA concentration in maternal plasma, f, was calculated from total reads as per the equation f = 2p/(p + q), where p is the number of sequence reads of the paternally inherited fetal allele and q is the number of sequence reads of the antithetical allele of the mother, which is shared by the maternal and fetal genomes [8,18].

## 3. Results

### 3.1. Range and Linearity

**Figure 1:**
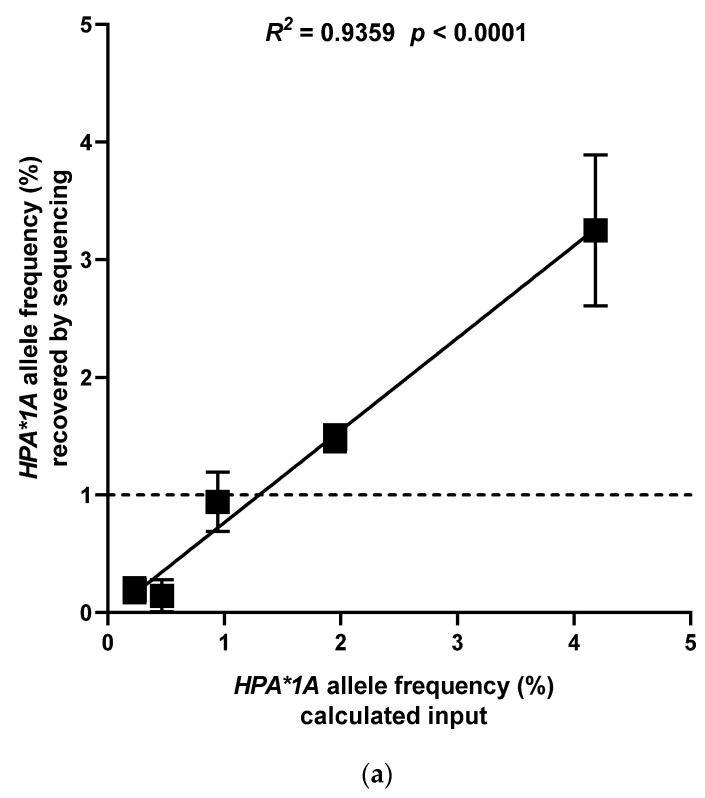

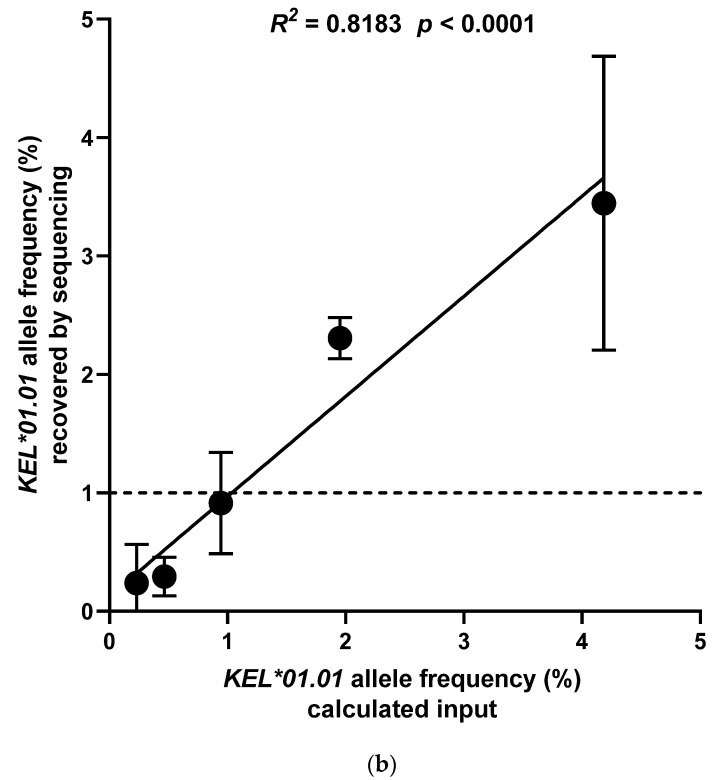
(**a**) One HPA*1A and KEL*01.01-positive cell-free plasma sample was serially diluted in HPA*1A and KEL*01.01-negative cell-free plasma (*N* = 1; 3 technical replicates). Y-axis shows determined HPA*1A allele frequencies after cell-free DNA extraction and targeted next-generation sequencing. X-axis shows the calculated HPA*1A allele frequencies obtained by serial dilution. Linear regression *R*^2^ = 0.9359, *p* < 0.0001. Dashed line: predefined cut-off for paternally inherited alleles (1%). (**b**) One HPA*1A and KEL*01.01-positive cell-free plasma sample was serially diluted in HPA*1A and KEL*01.01-negative cell-free plasma (*N* = 1; 3 technical replicates). Y-axis shows determined KEL*01.01 allele frequencies after cell-free DNA extraction and targeted next-generation sequencing. X-axis shows the calculated KEL*01.01 allele frequencies obtained by serial dilution. Linear regression, *R*^2^ = 0.8183, *p* < 0.0001. Dashed line: predefined cut-off for paternally inherited alleles (1%).

To determine the range and linearity, HPA-1ab- and Kk-positive donor plasma (mimicking the fetus) was added to Streck-BCT-collected donor plasma from an HPA-1bb- and kk(Cellano)-positive donor (mimicking the mother) in multiple dilution steps (5, 10, 20, 40, and 80-fold dilutions). Each dilution was tested in three PCR replicates, and the concentration of cfDNA in the donor plasma was measured. A correction factor was applied to normalize the results, as detailed in Supplementary Table S2. Signals were detected for both SNPs up to a 20-fold dilution (1%) within the linear range. In both dilution series, recovery was adequate (*p* < 0.0001), as shown in Figure 1a,b.

### 3.2. False-Positive Antithetic Alleles

**Figure 2:**
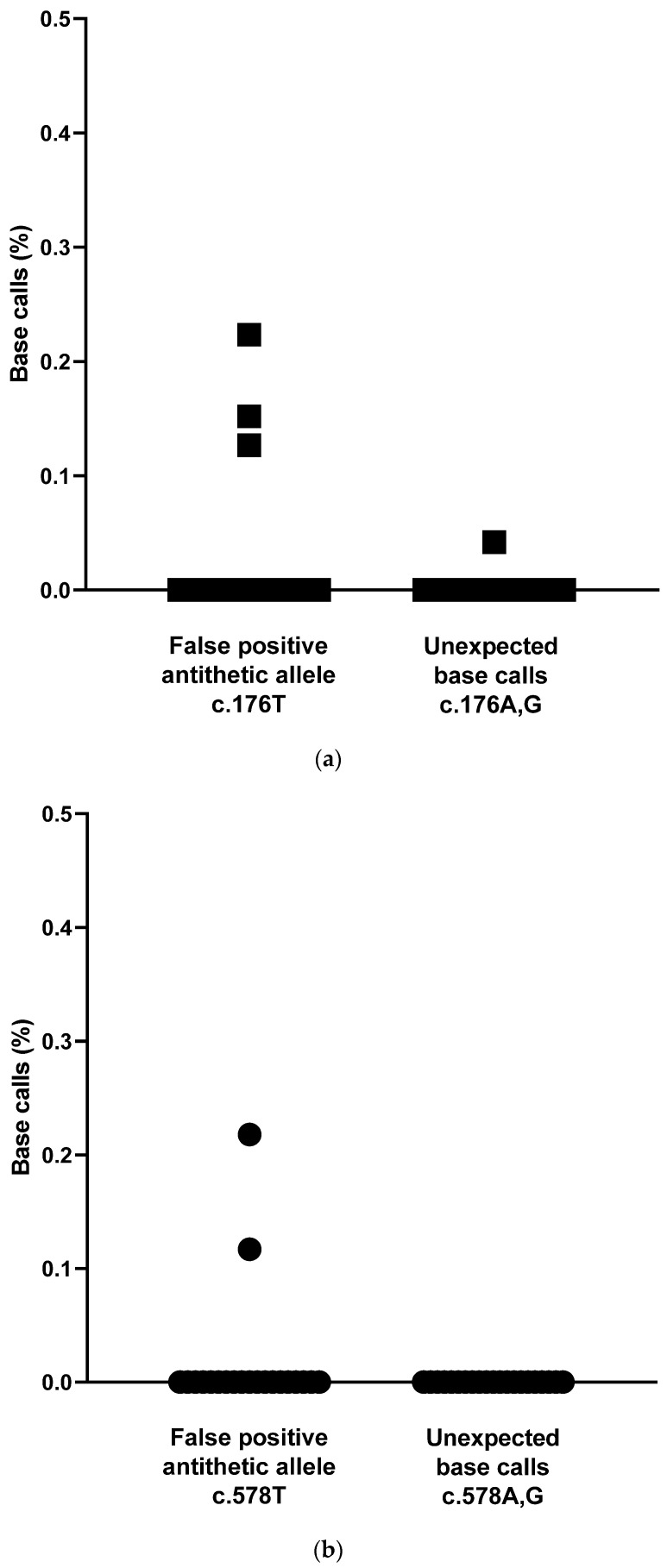
(**a**). Targeted next-generation sequencing of cell-free plasma DNA from HPA*1A negative blood donors (*N* = 21). False-positive base calls in % for HPA*1 SNP (rs5918, *ITGB3* c.176T>C) after correction for technical errors using unique molecular identifiers. There were 0.0000% (median, 95% *CI*: 0.0000, 0.0000) false-positive base calls for the HPA*1A allele (c.176T) and 0.0000% (median, 95% *CI*: 0.0000, 0.0000) false-positive base calls for unexpected alleles (c.176A,G). (**b**). Targeted next-generation sequencing of cell-free plasma DNA from KEL*01.01-negative blood donors (*N* = 21). False-positive base calls in % for KEL*01.01 SNP (rs8176058, *KEL* c.578T>C) after correction for technical errors using unique molecular identifiers. There were 0.0000% (median, 95% *CI*: 0.0000, 0.0000) false-positive base calls for the KEL*01.01 allele (c.578T) and 0.0000% (median, 95% *CI*: 0.0000, 0.0000) false-positive base calls for unexpected alleles (c.578A/G).

To detect false-positive HPA*1A and KEL*01.01 base-call frequencies, cell-free DNA was obtained from HPA*1A- and KEL*01.01-negative control plasma samples (*N* = 21). After correction for technical errors using unique molecular identifiers, the frequencies of the false-positive antithetic allele (FPATA) T for SNP rs5918 were obtained with a median (95% CI) of 0.0000% (0.0000, 0.0000). Unexpected base calls (UEBCs) for SNP rs5918 were detected with a median frequency of 0.0000% (95% CI: 0.0000, 0.0000), as shown in Figure 2a.

The frequencies of the false-positive antithetic allele (FPATA) T for SNP rs8176058, after correction for technical errors using unique molecular identifiers, were calculated with a median (95% *CI*) of 0.0000% (0.0000, 0.0000), as illustrated in Figure 2b. UEBCs A and G for SNP rs8176058 were detected with a median frequency of 0.0000% (95% CI 0.0000, 0.0000).

### 3.3. Limit of Detection (LOD) and Limit of Quantification (LOQ)

The LOD and LOQ were determined by analyzing cfDNA from HPA-1bb- and kk-positive plasma from blood donors (*N* = 21), as shown in Table 1. The limit of detection was calculated using the mean plus 3 times the value of the standard deviation, and the limit of quantification using 10 times the value of the standard deviation. For HPA*1A, the LOD was 0.2057% (mean + 3 SD) and the LOQ was 0.6298% (mean + 10 SD). For KEL*01.01, the LOD was 0.1706% (mean + 3 SD) and the LOQ was 0.5314% (mean + 10 SD).

### 3.4. Baseline Characteristics of NIPT Results in Diagnostic Samples

The method was validated using diagnostic samples from pregnant women. Table 2 provides an overview (*N* = 23). The mean gestational age (GA) of pregnant women was 22 weeks (range, 13 to 30 weeks). HPA-1a and K incompatibilities were the most commonly requested analyses. The paternally inherited fetal allele frequencies of positive test results (*n* = 14) were in the estimated range for the week of gestation [19]; conversely, the paternally inherited fetal allele frequencies of negative test results (*n* = 10) were zero or below 0.2% when using the method. Samples for confirmatory typing of the newborn were obtained in most cases (71.43%, *n* = 15 of 21).

### 3.5. Detection of Paternally Inherited ITGB3 (HPA-1a) and KEL

The value of paternally inherited *ITGB3* and *KEL* gene sequences was calculated. Of the 23 diagnostic samples analyzed, fetal alleles were identified in the plasma of 7 pregnant women with seven HPA-1ab fetuses, with a median (95% CI) of 5.41% (3.24–9.51). In samples from five KEL*02 pregnant women, paternally inherited KEL*01.01 sequences were detected with a median (95% CI) of 5.85% (3.15–8.99).

### 3.6. False-Positive Antithetic Allele Frequencies of ITGB3 and KEL

False-positive HPA*1A antithetic allele frequencies were detected in the plasma of two pregnant women with HPA-1bb fetuses with a median (95% CI) of 0.0805% (0.0000, 0.1610, *n* = 2). Similarly, false-positive KEL*01.01 antithetical allele frequencies were found in the plasma of 16 pregnant women with KEL*02 fetuses, with a median (95% CI) of 0.0000% (0.0000, 0.0000; *n* = 16).

### 3.7. Inter-Assay Variability

**Figure 3:**
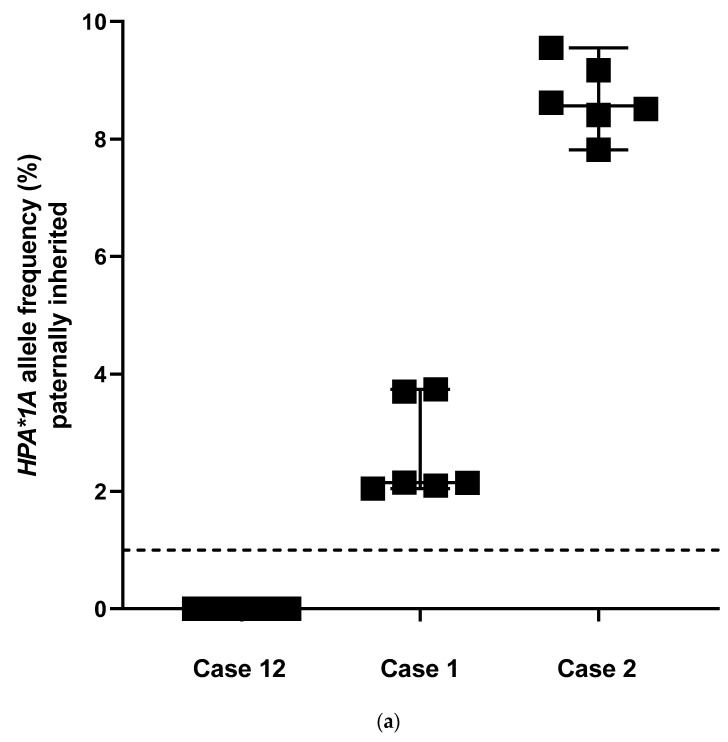

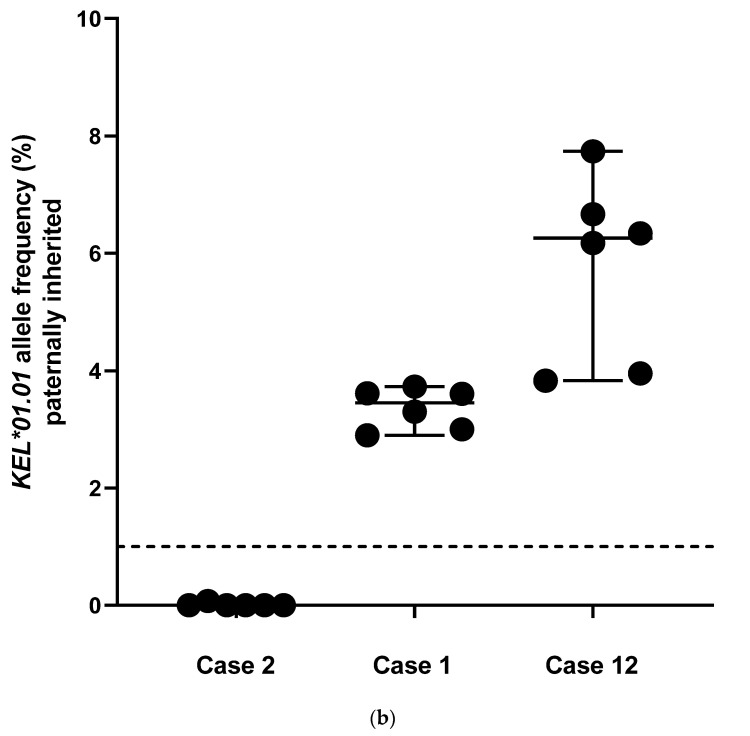
(**a**). Inter-assay variation in the quantification of paternally inherited HPA*1A allele frequencies using targeted next-generation sequencing. Plasma samples of three pregnant women with negative, low, and high counts of fetal HPA*1A alleles. Of each plasma sample, three independently prepared libraries were sequenced in two different runs. Case 12 (negative) median 0.00%, 95% *CI*: 0.00, 0.00; Case 1 (low) median 2.15%, 95% *CI*: 2.05, 3.74; Case 2 (high) median 8.57%, 95% *CI*: 7.82, 9.55. Dashed line: predefined cut-off for paternally inherited alleles (1%). (**b**). Inter-assay variation in the quantification of paternally inherited KEL*01.01 allele frequencies using targeted next-generation sequencing. Plasma samples of three pregnant women with negative, low, and high counts of KEL*01.01 alleles. Of each plasma sample, three independently prepared libraries were sequenced in two different runs. Case 2 (negative) median 0.00%, 95% *CI*: 0.00, 0.08; Case 1 (low) median 3.46%, 95% *CI*: 2.90, 3.73; Case 12 (high) median 6.26%, 95% *CI*: 3.83, 7.74. Dashed line: predefined cut-off for paternally inherited alleles (1%).

Plasma samples from three different HPA-1bb, kk women (cases 1, 2 and 12) were analyzed with three replicates in two sequence runs, the results of which are shown in Figure 3. As expected, no signals were detected in case 12, which carried an HPA-1bb fetus, with a median (95% CI) of 0.00% (0.00, 0.00). In the other two samples (cases 1 and 2), the HPA*1A allele inherited from the father was detected, with median values of 2.15% (95% CI: 2.05–3.74; case 1) and 8.57% (95% CI: 7.82–9.55; case 2), respectively (Figure 3a). For KEL*01.01 (rs8176058, c.578C>T), the following frequencies of the paternally inherited KEL*01.01 allele (T for the SNP rs8176058) were absent in case 2, with a median (95% CI) of 0.00% (0.00–0.08). This allele was detected in case 1, with a median (95% CI) of 3.46% (2.90–3.73), and in case 12, with a median (95% CI) of 6.26% (3.83–7.74) (Figure 3b).

### 3.8. Quantification of Fetal Fraction Using SNPs

**Figure 4:**
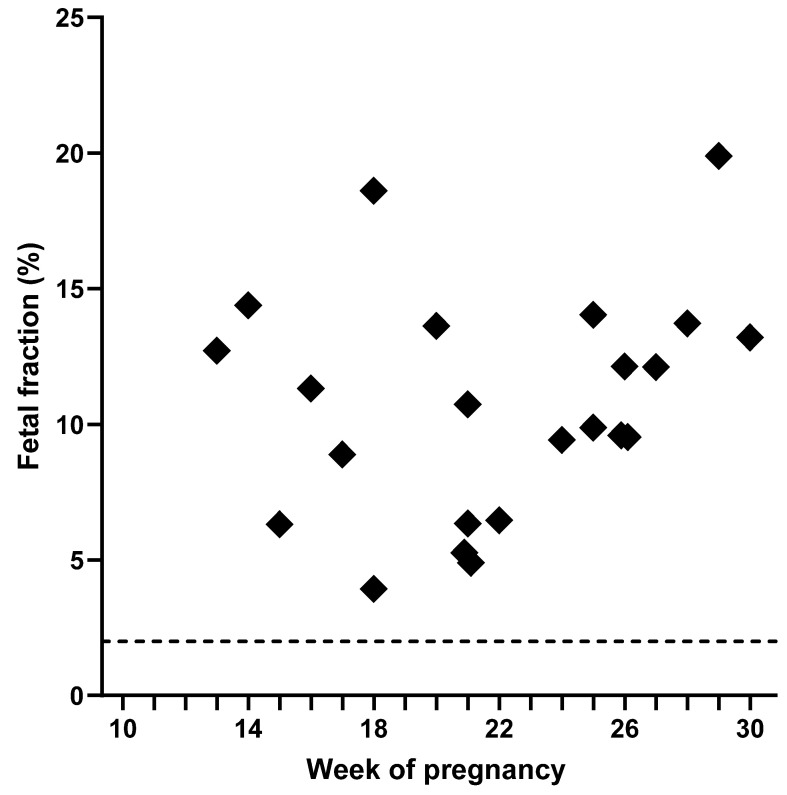
Targeted next-generation sequencing of cell-free DNA from 23 plasma samples of 21 pregnant women (*N* = 23). Each data point represents the calculated fetal fraction, median 11.03% (95% *CI*: 8.89, 13.20). Dashed line: predefined cut-off value for the fetal fraction (2%).

Figure 4 shows the calculated fetal fractions assessed with diagnostic samples, with a median (95% CI) of 11.03% (8.89–13.20, *N* = 23). In general, a high fetal fraction was observed using this approach.

## 4. Discussion

In comparison to the high number of samples processed for *RHD* screening, only limited sample numbers are available for other potentially incompatible fetal antigens such as, HPA-1a or K. No WHO-standard DNA is available for antigens other than Rhesus D. In order to validate the NGS method according to the recommendations available for fetal *RHD* screening tests [6], we used “incompatible” plasma dilutions to calculate the LOD, LOQ, false-positive antithetic allele quantification, range, and linearity of our method. Streck tubes were used for the present study to prevent an increase in maternal cfDNA, a cautionary measure on which most groups seem to agree [6]. Data obtained with this approach show that NGS for non-invasive fetal blood group genotyping is accurate and reliable, and that plasma dilution is a helpful and feasible approach for validating this method.

Our approach using healthy blood donors revealed frequencies for false-positive antithetic alleles between 0.0159% mean (range, 0.0000–0.2179%) and 0.0239% mean (range, 0.0000–0.2235%) and showed good agreement with the results of the negative diagnostic samples (Table 2) with absent paternal allele (maximum 0.16%). False-positive signals in NGS are not based on systematic errors, rather, they reflect contamination during library preparation or sequencing. Other groups have published comparable figures: Rieneck et al. calculated a threshold value of 0.0661% for *KEL* [20], and Orzińska et al. showed that the percentage of reads for blood group alleles in the plasma of pregnant women with antigen-negative fetuses ranged between 0% and 0.87%. In a case of a KEL*02/KEL*02 fetus, 0.19% false-positive KEL*01.01 reads were detected [11]. False-positive events are clearly discriminated from values of confirmed positive samples (signal-noise ratio). We are well aware that we examined cfDNA from healthy blood donors and that fetal cell-free DNA fragments are shorter. This could theoretically affect the validity of our calculations. However, we chose an amplicon design with very short insert sizes, which performs well with fetal DNA fragments (which are approximately 20 bp shorter than cfDNA fragments from mothers or, in our case, blood donors).

In addition to spiking antigen-positive blood donor plasma into antigen-negative plasma, we also analyzed samples from pregnant women. The allele frequency for antigen-positive fetuses ranged from 3.24% to 9.51% for HPA-1a and from 3.15% to 7.74% for KEL*01. For antigen-negative fetuses, the calculated fetal fraction from SNPs included as an internal control was at least 13.63% for HPA-1a and at least 5.27% for K, which compares to published data; Alford et al. reported a fetal fraction of 8.8% (range 1.5–37.3%) [21].

As demonstrated by others, a threshold value for the acceptable fetal fraction of cell-free DNA should be set to avoid false-negative results [14]. Our data show that for HPA-1a and K, a threshold of 1% (frequency of paternal alleles ≥ 1%; fetal fraction ≥ 2%) appears to be feasible. This threshold may not apply for other genetic variants or other methodological approaches.

## 5. Conclusions

NGS is a reliable method with high sensitivity. It is feasible to perform in-house validation with healthy donors in order to determine LOD, LOQ, and analytical sensitivity. The threshold for paternally inherited fetal HPA*1A and KEL*01.01 alleles could be set at 1% (i.e., at 2% fetal fraction). Internal controls for assessing the fetal fraction are useful when the implicated antigen is not amplified.

## Figures and Tables

**Table 1:** Limit of detection and limit of quantification determination using HPA*1A and KEL*01.01 negative controls (*N* = 21).

False-Positive Antithetic Allele Frequency of Negative Controls	HPA*1A (rs5918, c.176T>C) % T	KEL*01.01 (rs8176058, c.578C>T) % T	Unexpected Base Calls of Negative Controls	HPA*1A (rs5918, c.176T>C) % A,G	KEL*01.01 (rs8176058, c.578C>T) % A,G
Mean	0.0239	0.0159	Mean	0.0020	0.0000
Standard deviation	0.0606	0.0516	Standard deviation	0.0090	0.0000
3× Standard deviation	0.1818	0.1547			
Limit of detection (Mean + 3SD)	0.2057	0.1706			
10× Standard deviation	0.6059	0.5155			
Limit of quantification (Mean + 10SD)	0.6298	0.5314			

**Table 2:** Non-invasive prenatal diagnostic blood samples (*N* = 23) from 21 pregnant women tested by the targeted sequencing assay (*n* = 24).

Case	Week of Pregnancy	Maternal Alloantibody	SNP ID	Fetal Allele Frequency (%)	Total SNP-Based Fetal Fraction (%)	Non-Invasive Prenatal Test Result	Postnatal Confirmatory Result
1	15	HPA-1a	rs5918	3.74	6.31	positive	yes
2	18	HPA-1a	rs5918	9.51	18.61	positive	yes
3	26	HPA-1a	rs5918	5.72	9.59	positive	n.a.
4	26	HPA-1a	rs5918	3.24	9.53	positive	yes
5	26	HPA-1a	rs5918	4.04	12.13	positive	yes
6	20	HPA-1a	rs5918	0.00	13.63	negative	yes
7	29	HPA-1a	rs5918	0.16	19.89	negative	n.a.

8	18	HPA-5b	rs1801106	1.90	3.93	positive	yes
8 *	21	HPA-5b	rs1801106	2.10	4.89	positive	yes
9	25	HPA-5b	rs1801106	3.84	9.88	positive	yes
10	28	HPA-5b	rs1801106	0.08	13.72	negative	n.a.

11	24	K	rs8176058	3.15	9.43	positive	yes
12	25	K	rs8176058	7.74	14.04	positive	yes
13	27	K	rs8176058	5.85	12.11	positive	n.a.
14	13	K	rs8176058	0.00	12.72	negative	yes
15	14	K	rs8176058	0.00	14.39	negative	yes
15 *	21	K	rs8176058	0.00	6.35	negative	yes
16	21	K	rs8176058	0.00	5.27	negative	yes

17	16	Jk(a)	rs1058396	6.97	11.32	positive	yes
18	17	Jk(a)	rs1058396	2.48	8.89	positive	n.a.
14 #	13	Jk(a)	rs1058396	0.00	12.72	negative	yes

19	22	E	rs609320	2.38	6.46	positive	yes
20	30	E	rs609320	0.00	13.20	negative	n.a.

21	21	S	rs7683365	0.00	10.74	negative	yes

*: repeated testing; n.a.: not analyzed; #: sample with two incompatibilities.

## Data Availability

The data that support the findings of this study are available from the corresponding author upon reasonable request.

## Supplementary Materials

The following supporting information can be downloaded at: https://www.mdpi.com/article/10.3390/jcm14196812/s1, Supplementary Table S1: Targeted SNPs and exonic regions encoding common blood group phenotypes of PLTs and RBCs using next-generation sequencing; Supplementary Table S2: Input calculation of *HPA*1A* and *KEL*01.01* allele frequencies using cell free DNA from serial *HPA*1A* and *KEL*01.01* positive plasma dilutions.

## Abbreviations

The following abbreviations are used in this manuscript:

AF	Allele frequency
Bp	Base pairs
ccfDNA	Circulating cell-free DNA
cfDNA	Cell-free Deoxyribonucleic Acid
cffDNA	Cell-free fetal Deoxyribonucleic Acid
ddPCR	droplet digital PCR
HDFN	Hemolytic disease of the fetus and newborn
FNAIT	Fetal and neonatal alloimmune thrombocytopenia
FPATA	False-positive antithetic allele
GA	Gestational age
LOD	Limit of detection
LOQ	Limit of quantification
NGS	Next-Generation Sequencing
NIPD	Non-invasive prenatal diagnostic
NIPDT	Non-invasive prenatal diagnostic test
PGM	Personal Genome Machine
PLT	Platelet
RBC	Red Blood Cell
RT-PCR	Reverse Transcription PCR
SNP	Single-Nucleotide Polymorphism
UEBC	Unexpected base calls
