# Supplementary material for: A Validation Approach for Determining Fetal Blood Groups Non-Invasively by High-Sensitive Next-Generation Sequencing
Source: J Clin Med. 2025 Sep 26;14(19):6812. doi: 10.3390/jcm14196812 (PMC12524485; doi:10.3390/jcm14196812)
Supplement: Supplementary file 1 [file jcm-14-06812-s001.zip › jcm-3861376-supplementary.pdf]

Supplementary Table 1. Targeted SNPs and exonic regions encoding common blood group phenotypes of PLTs and RBCs using next-generation sequencing.

System/target	*Gene*	SNP ID	Phenotype	Insertsize
HPA-1	*ITGB3*	rs5918	HPA-1a/b	76
HPA-4	*ITGB3*	rs5917	HPA-4a/b	74
HPA-5	*ITGA2*	rs1801106	HPA-5a/b	62
HPA-15	*CD109*	rs10455097	HPA-15a/b	68
RHD Exon 4	*RHD*	rs569974439	D	74
RHD Exon5 (Pos 697)	*RHD*	rs1053359	D	58
RHD Exon 7	*RHD*	rs138235491	D	44
RHCE Exon 1	*RHCE*	rs586178	C/c	49
RHCE Exon 5	*RHCE*	rs609320	E/e	65
RHCE Exon 2	*RHCE*	rs676785	C/c	80
C weak	*RHCE*	rs138268848	Wildtyp	50
Kell/cellano	*KEL*	rs3757853	K/k	72
Kell/cellano	*KEL*	rs8176044	K/k	44
Kell/cellano	*KEL*	rs8176058	K/k	58
Duffy	*DARC*	rs12075	Fya/b	78
Kidd	*SLC14A1*	rs1058396	Jka/b	69
MNS	*GYPB*	rs7683365	S/s	74
CO	*AQP1*	rs28362692	Co-a/b	60
LU	*BCAM*	rs28399653	Lu-a/b	82
DI	*SLC4A1*	rs75731670	Wr-a/b	47
SRY	*SRY*	rs11575897	Y	78
SNP #1	*ATP13A4*	rs6444724		69
SNP #2	*PALLD*	rs6811238		64
SNP #3	*ADAMTS2*	rs338882		67
SNP #4	*PTN*	rs321198		71
SNP #5	*TRDMT1*	rs3780962		70
SNP #6	*P3H3*	rs2269355		47
SNP#7	*RAB31*	rs9951171		76
SNP #8	*LARGE*	rs987640		71
SNP#9	*FZD3*	rs10092491		67
SNP #10	*LINGO2*	rs7041158		77
SNP#11	*CADM1*	rs10488710		75
SNP #12	*no gene name*	rs1821380		80
SNP #13	*UBAC2*	rs1058083		83
SNP #14	*HSPA12A*	rs740598		73
SNP #15	*no gene name*	rs4530059		72
SNP#16	*CERKL*	rs12997453		65
SNP#17	*MROH6*	rs4606077		81
SNP#18	*SYNE1*	rs214955		74
SNP#19	*LOC100506532*	rs10776839		82
SNP #20	*LOC105373422*	rs1109037		74
SNP#21	*no gene name*	rs576261		75
SNP#22	*TXNRD2*	rs5746846		80
SNP#23	*SASH1*	rs2272998		67
SNP #24	*no gene name*	rs4288409		78
SNP #25	*NBR2*	rs2175957		70

Supplementary Table 2. Input calculation of *HPA*1A* and *KEL*01.01* allele frequencies using cell free DNA from serial *HPA*1A* and *KEL*01.01* positive plasma dilutions.

	Heterozygous *HPA*1A* and *KEL*01.01* Positive Plasma	Homozygous *HPA*1A* and *KEL*01.01* Negative Plasma	
**Dilution 1:5**	Undiluted	Undiluted	Dilution 1:5
Plasma DNA Concentration (pg/µl)	232.00	635.00	
Plasma Volume in Dilution (ml)	1.30	5.20	6.50
DNA Amount in 1:5 Dilution (ng)	301.60	3302.00	3603.60
Total Allele Frequency %	8.37	91.63	100.00
Heterozygous Allele Frequency %	**4.18**		
**Dilution 1:10**	Undiluted	Undiluted	Dilution 1:10
Plasma DNA Concentration (pg/µl)	232.00	635.00	
Plasma Volume in Dilution (ml)	1.30	11.70	13.00
DNA Amount in 1:10 Dilution (ng)	301.60	7429.50	7731.10
Total Allele Frequency %	3.90	96.10	100.00
Heterozygous Allele Frequency %	**1.95**		
**Dilution 1:20**	1:10 Dilution	Undiluted	Dilution 1:20
DNA Amount in 1:10 Dilution (ng)			7731.10
Plasma DNA Concentration (pg/µl)		635.00	
Plasma Volume in Dilution (ml)	6.50	6.50	13.00
DNA Amount in 1:20 Dilution (ng)	3865.55	4127.50	7993.05
DNA Concentration in Positive Plasma Dil	150.80		
Total Allele Frequency %	1.89		
Heterozygous Allele Frequency %	**0.94**		
**Dilution 1:40**	1:20 Dilution	Undiluted	Dilution 1:40
DNA Amount in 1:20 Dilution (ng)			7993.05
Plasma DNA Concentration (pg/µl)		635.00	
Volume Plasma in Dilution (ml)	6.50	6.50	13.00
DNA Amount in 1:40 Dilution (ng)	3996.53	4127.50	8124.03
DNA Concentration in Positive Plasma Dil	75.40		
Total Allele Frequency %	0.93		
Heterozygous Allele Frequency %	**0.46**		
**Dilution 1:80**	1:40 Dilution	Undiluted	Dilution 1:80
DNA Amount in 1:40 Dilution (ng)			8124.03
Plasma DNA Concentration (pg/µl)		635.00	
Volume Plasma in Dilution (ml)	6.50	6.50	13.00
DNA Amount in 1:80 Dilution (ng)	4062.02	4127.50	8189.52
DNA Concentration in Positive Plasma Dil	37.70		
Total Allele Frequency %	0.46		
Heterozygous Allele Frequency %	**0.23**		
